# Pre- and postsurgical anxiety and body perception: the significance of cognitive-behavioral therapy in scoliosis adolescents

**DOI:** 10.1186/s12891-026-09645-9

**Published:** 2026-02-25

**Authors:** Ewa Misterska, Marek Tomaszewski, Patrycja Marcinak-Stępak, Maciej Głowacki

**Affiliations:** 1 Department of Mental Health, Chair of Psychiatry, Poznan University of Medical Sciences, ul. Szpitalna 27/33, Poznan, 60-355 Poland; 2 Department of Spine Disorders and Pediatric Orthopedics, Poznan University of Medical Sciences, ul. 28 Czerwca 1956 135/147, Poznan, 61-545 Poland; 3 Department of Pediatric Oncology, Hematology and Transplantology, Institute of Pediatrics, Poznań University of Medical Sciences, ul. Szpitalna 27/33, Poznan, 60-355 Poland; 4 Department of Pediatric Orthopedics and Traumatology, Poznan University of Medical Sciences, ul. 28 Czerwca 1956 135/147, Poznan, 61-545 Poland

**Keywords:** Adolescent idiopathic scoliosis, Anxiety, Body image, CBT, Surgical treatment

## Abstract

Background. Spinal disfigurement is one of AIS patients’ greatest concerns and the primary objective of treatment. No group has ever evaluated the anxiety and body image disturbances longitudinally in three time points in the course of scoliosis surgical treatment, following the cognitive-behavioral therapy (CBT). The aim of this study was a longitudinal analysis of changes in anxiety levels and to determine their associations with a perception of trunk deformity in females with adolescent idiopathic scoliosis (AIS) who either did or did not undergo CBT. Methods. The study design comprised of three questionnaire assessments, with the evaluations taking place before and after surgical treatment during the hospital stay and in a minimum 6-months follow-up postsurgery. A sample of healthy females was also included in the analyses for comparative purposes. Thirty-six AIS females in total (18 received CBT and 18 did not) filled in the Polish trait version of the Spielberger’s Anxiety Inventory for Children (STAIC-trait), Trunk Appearance Perception Scale (TAPS). The Trunk Aesthetic Clinical Evaluation (TRACE) was completed by the doctors involved and patients pre-and postsurgery. Results. In regard to TAPS, the differences between all assessments were significant for both scoliosis groups, in favor, as expected, of postsurgical results. However, the improvement of the perception of patients’ body shape remained significant in the follow-up only in the CBT scoliosis sample (*p* = 0.018). Referring to TRACE-patients’ and TRACE-doctors’ results agreement, there was no concurrence between them (results of Kendall’s coefficients of concordance appeared insignificant). Conclusions. AIS patients’ anxiety levels are stable, independent of received therapeutic support. Clinicians need to be aware of how patients’ appearance-specific cognitions might be associated with levels of anxiety and the supporting role of psychotherapy in the further stabilization of patients’ improvement of body shape perception following scoliosis surgical treatment.

## Background

Adolescent idiopathic scoliosis (AIS), defined as a three-dimensional spine and trunk deformity, appearing in otherwise healthy subjects, exhibits complex relations with various forms of psychopathology and personal well-being. The majority of conducted research has documented a higher proportion of different psychological disturbances (e.g., self-criticism, body image disturbances (BID), low self-esteem) and mental disorders (e.g., anxiety) among AIS, compared to healthy controls [1].

In addition to standardized psychopathological disturbances following AIS treatment, some studies have also shown a lower body image that may persist for several years in surgical patients [2]. Other studies focused on anxiety related to AIS [3]. Most of the research referred to focused on spinal surgery- and scoliosis-related anxiety. Specifically, that anxiety and pain were considered major concerns for children who underwent surgery. Hawes [4] indicated AIS-related anxiety results from uncertainty about whether the spinal deformity and its symptoms will progress. Misterska et al. [5] found that surgically treated AIS patients exhibited higher anxiety compared with conservatively treated patients and healthy controls. In a longitudinal study of conservatively treated AIS patients [6], it was highlighted that patients’ emotional responses during ongoing brace treatment warrant attention, while their perception of spinal deformity generally remained stable over time. Recently, Rullander et al. [7] investigated stress symptoms among adolescents before and following scoliosis surgery; preoperative anger, social problems and attention deficits correlated significantly with postoperative pain at follow-up. Furthermore, Choate [8] emphasized the role of appearance-specific cognitions in influencing levels of emotional tension.

Concerning the goals of psychological support for patients undergoing spinal surgery, coping with anxiety and pain must be highlighted as these are key concerns for such patients [9, 10]. Brief interventions tailored to specific patient populations may provide effective coping strategies for young adolescents [11].

Since spinal disfigurement is one of their greatest concerns and the primary objective of treatment, we believe the emotional state of AIS females may be related to patients’ concerns and perceptions of body appearance. It is important to distinguish between related but conceptually different constructs in adolescents with idiopathic scoliosis. Body image disturbance refers to a broader psychological construct reflecting negative thoughts, emotions, and dissatisfaction with one’s body appearance. In contrast, subjective perception of trunk deformity reflects how patients visually and cognitively perceive the severity and asymmetry of their own trunk, while aesthetic evaluation of scoliosis represents an external, clinician-based assessment of visible trunk deformity. Previous studies have shown that these domains are correlated but not interchangeable, and each may contribute differently to psychological well-being and quality of life in adolescents with scoliosis [12]. Therefore, the combined use of patient-reported perception (Trunk Appearance Perception Scale, TAPS) [13, 14] and clinician-rated aesthetic assessment (Trunk Aesthetic Clinical Evaluation, TRACE) [15] allows a more comprehensive evaluation of both subjective and objective aspects of trunk appearance.

Therefore, the aim of this study was a prospective analysis of changes in anxiety levels and to determine their associations with a longitudinal subjective assessment of trunk deformity in females with AIS who either did or did not undergo cognitive-behavioral therapy (CBT). The CBT intervention was aimed at supporting AIS patients in accepting their actual body shape, changing their desired body shape in a more realistic manner and in feeling positive about the cosmetic results of surgical treatment. In addition, subjects’ aesthetic body perception will be compared to their doctors’ evaluation made by the same assessment tool. A sample of healthy females will also be added to analyses for comparative purposes.

## Methods

### Study design

The study employed a combined longitudinal and cross-sectional design. The longitudinal component involved three assessment points: (1) preoperative evaluation (T1) conducted at the Department of Pediatric Orthopedics and Traumatology and the Department of Spine Disorders and Pediatric Orthopedics, Poznan University of Medical Sciences, Poland; (2) postoperative assessment (T2), performed within two weeks after surgery at the same departments; and (3) follow-up evaluation (T3), conducted at least six months post-surgery, once spinal fusion had stabilized and natural compensatory mechanisms had developed in the region affected by the procedure.

The cross-sectional component aimed to examine differences in anxiety levels and body image perception among three groups: AIS patients who received a psychotherapeutic intervention (CBT scoliosis group), AIS patients who did not receive such intervention (control scoliosis group), and a sample of healthy female participants.

### Recruitment to the study

Participant recruitment for the scoliosis groups followed the inclusion and exclusion criteria described in Misterska et al. [16], focusing on females aged 12–18 years with thoracic AIS scheduled for posterior spinal fusion. Detailed information about the study protocol was provided to participants and their parents, and written informed consent was obtained. All surgeries were performed by the study authors using hybrid instrumentation with hooks and screws, with no postoperative complications affecting outcomes. The healthy female control group was recruited from randomly selected schools, with students and their parents receiving information about the study and providing written consent.

Inclusion criteria for the healthy female group were: female sex, age 12–18 years, and absence of scoliosis or other spinal deformities, as well as no serious medical conditions. Suspected scoliosis was assessed using the Adams forward bend test, following the procedure described by Santos et al. [17]. The evaluator, a pediatric orthopedist and study author (MG), observed the symmetry of the thoracic and lumbar spine during trunk flexion. Gibbosity was defined as localized spinal over-curvature, and the test outcome indicated either suspicion or absence of scoliosis [17]. Each participant received a record of their test results.

Initially, 40 scoliosis patients met the eligibility criteria. Two declined participation, and two were excluded due to mental impairment, resulting in 36 participants (18 in the CBT scoliosis group and 18 in the control group) completing T1 and T2 assessments. At T3, follow-up data were available for 25 patients, as 4 from the CBT group and 7 from the control group could not be contacted.

For the healthy female group, 29 individuals were initially eligible. Ten declined participation, and one was excluded following clinical detection of scoliosis, leaving 18 participants included in the study.

### Ethical issues

The study was conducted in accordance with the Declaration of Helsinki and received approval from the Bioethical Commission at Poznan University of Medical Sciences (Nos. 695/18, 800/22) and the Center for Safety Research at the University of Security in Poznan (Nos. 001/2018, 001/2022).

### The content of CBT intervention

The CBT intervention was planned pre- and postoperatively, during the patients’ stay in the hospital ward and was administered by a CBT psychotherapist. The number of planned therapeutic sessions was designed to optimize attendance with the time required to develop the needed skills and depended on the patient’s needs. After hospital discharge, continuity of care and CBT support through telecommunication devices (e-mail, chat, and telephone as preferred) was offered to the CBT scoliosis sample.

The intervention content was modeled after the CBT body image therapy of Thompson [18]. The presurgical sessions included discussing the emotions and beliefs that are involved in negative body image and the consequences of behavior that negative body image promotes, such as dieting, checking, and avoidance behaviors. Participants also discussed activators of negative body image, such as sociocultural, peer, and familial and health-related factors. In addition, a presurgery session included writing down and discussing patients’ appearance-preoccupying rituals. During another presurgical session, participants were asked to write down and discuss what they believe would happen if their distressing body part was openly revealed. Participants were also asked to discuss the behaviors they used to compensate for their body image, if any.

The content of the postoperative sessions were strongly dependent on the needs of the individual patient. It was explained that negative thoughts toward one’s silhouette are learned and, therefore, can be unlearned, specifically bearing in mind meaningful visual improvement of body shape following surgical treatment. Firstly, patients were taught relaxation techniques, including deep breathing and progressive muscle relaxation. Secondly, participants were also taught how to refrain from critical self-talk by substituting more objective sensory descriptions of their corrected body parts. The therapist modelled corrective body talk with examples provided by the patient, and then discussed its benefits. Furthermore, thought stopping, paired with relaxation, were introduced as a way to extinguish subjective distress [18]. To summarize, the CBT intervention was aimed at supporting AIS patients in accepting their actual body shape, changing their desired body shape in a more realistic manner and in feeling positive about the cosmetic results of surgical treatment.

### Analyzed data

Data available on request.

#### Socio-demographic, activity-related, clinical and radiological data

Data collected included participants’ age, residence, school, weight, height, and any chronic conditions. Information on physical activity prior to surgical treatment was also obtained, including participation in school physical education (yes/no), engagement in recreational sports (yes/no), and, if applicable, weekly hours of practice.

For the scoliosis groups, clinical and radiological assessments were performed at T1 and T2. Standard anterior–posterior and lateral spine X-rays were obtained, and the following parameters were analyzed: Cobb angle of the main curve, thoracic kyphosis, lumbar lordosis, apical translation relative to the central sacral vertical line (CSVL) as per the Harms Study Group, Lenke classification, main curve location, and percentage of surgical correction. Additional data included age at scoliosis diagnosis, family history of scoliosis, and duration of any brace treatment (in months).

#### Perception of body deformity

Adolescents’ perception of trunk deformity was assessed using the Polish version of the TAPS. The scale includes images of the trunk from three perspectives: posterior, forward-bending (Adams test), and frontal, with separate drawings for males and females. Each image is scored from 1 (minimal deformity) to 5 (maximum deformity), and the mean of the three scores is calculated to represent overall body deformity perception [13, 14].

The TRACE [15] as a no-cost routine clinical tool, was completed by the doctors pre- and postsurgery. However, both scoliosis samples (pre-, post-surgery and at follow-up) as well as the healthy female sample were also asked to complete this assessment for comparative purposes. Specifically, the comparison between clinician-rated and patient-rated TRACE scores enabled the evaluation of potential discrepancies in trunk deformity assessment. TRACE is based on four sub-scales: shoulders, scapulae and waist (which were already present in the Aesthetic Index (AI), and the hemithorax [15]. They correspond to Figs. 1, 2, 3 and 4, respectively. However, the scores for each sub-scale were changed with respect to the AI: shoulders now ranged from 0 to 3, waist from 0 to 4, scapulae from 0 to 2 and hemithorax from 0 to 2. From these sub-scales, TRACE was calculated, using the sum of the sub-scale scores to reach a 12-point scale [15].

#### Anxiety

Anxiety levels were evaluated using the Polish version of State-Trait Anxiety Inventory for Children (STAIC) [18]. The inventory includes separate scales for state and trait anxiety; only the trait scale (STAIC-trait) was used to capture more enduring anxiety characteristics. STAIC-trait comprises 20 items, with participants rating the frequency of anxiety symptoms (e.g., “I am scared,” “I feel troubled”) on a three-point scale: 1 = almost never, 2 = sometimes, 3 = often. A total trait anxiety score was calculated by summing all item ratings [19].

### Statistical methods

In terms of descriptive statistics of quantitative features, the following indicators were determined: mean, median, minimum and maximum values, standard deviation, 95% confidence intervals. In respect to qualitative features, we gave the number of units that belong to described categories of a given feature and respective percentages. Referring to longitudinal study design, to compare differences in results between the three time points, the repeated measures ANOVA, the Greenhouse-Geisser correction with multiple comparisons’ Tukey test or the Friedman test with Dunn Bonferroni multiple comparisons test were calculated. The Spearman’s rank correlation coefficient (rs) was used for calculating correlations between quantitative variables. Comparisons between two groups were performed using the t-test or the Mann-Whitney test. Comparisons between multiple groups were performed using the one-way AVOVA test, the F Welch test (with the Tukey multiple comparison test) or the Kruskal-Wallis test with the Dunn-Bonferroni multiple comparison test. The relationship between qualitative variables was tested using the chi2 test, the Fisher exact test or the Fisher-Freeman-Halton test. The agreement between TRACE completed by the doctors and scoliosis samples was calculated using Kendall’s W coefficient. The accepted border level of statistical significance was *p* = 0.05 and, therefore, any test results where the p value exceeded this level were treated as insignificant.

## Results

### Study sample characteristics

Table 1 presents detailed characteristics of study samples. Participants’ current age was 14.17 (SD 2.01), 14.50 (SD 1.50) and 13.56 (SD 1.15) in CBT scoliosis sample, control scoliosis samples and healthy females, respectively. Patients in both scoliosis samples had thoracic scoliosis. Fifteen patients (83.33%) in the group receiving therapy and 16 patients (88.89%) in the scoliosis control sample were treated conservatively prior to surgery for an average of 24.11 months (SD 20.20) and 35.06 months (SD 25.43), respectively. In the CBT scoliosis sample the average value of the Cobb’s angle in the main curve was 55.3 degrees (SD 9.7) at T1, whereas at T2 it was 23.22 degrees (SD 7.69). In the scoliosis control sample the mean of the Cobb angle at T1 was 62.16 degrees, whereas at T2 it was 24.27 degrees (SD 8.76). The mean percent of scoliosis correction was 60.50 degrees (SD 11.67) in the CBT group and 61.5 degrees (SD 11.46) in the scoliosis control sample.

**Table 1:** Socio-demographic, activity-related, clinical and radiological characteristics of study samples

	CBTSS			CSS			HFS			*p* value comparison CBTSS/ CSS/HFS
	Mean (SD)	Range (Min-Max)	*n* (%)	Mean (SD)	Range (Min-Max)	*n* (%)	Mean (SD)	Range (Min-Max)	*n* (%)	
Socio-demographic characteristics (CBTSS, CSS, HFS)										
Place of residence										*p* = 0.00002*
Rural	*p* = 0.060	-	3 (16.67)	-	-	7 (38.89)	-	-	0 (0)	
City below 25, 000 inhabitants	*p* = 0.207	-	8 (44.4)	-	-	2 (11.11)	-	-	3 (16.67)	
City between 25, 000 and 200, 000 inhabitants	-	-	4 (22.22)	-	-	7 (38.89)	-	-	1 (5.56)	
City over 200, 000 inhabitants	-	-	3 (16.67)	-	-	2 (11.11)	-	-	14 (77.77)	
School attended	-									
Elementary	-	-	9 (50.00)	-	-	9 (50.00)	-	-	15 (83.33)	*p* = 0.060
Secondary	-	-	9 (50.00)	-	-	9 (50.00)	-	-	3 (16.66)	
Current age [years]	14.17 (2.01)	12–17	-	14.50 (1.50)	12–17	-	13.56 (1.15)	12–16	-	*p* = 0.060
Age at scoliosis diagnosis [years]	9.9 (3.10)	5–14	-	10.11 (2.56)	5–14	-	-	-	-	*p* = 0.816
Weight [kg]	53.22 (9.61)	35–70	-	52.33 (11.01)	38–85	-	62.00 (15.71)	40–100	-	*p* = 0.086
Height [cm]	160.17 (6.40)	149–174	-	160.89 (5.99)	153–172	-	163.00 (6.75)	153–176	-	*p* = 0.392
Body Mass Index	20,63884 (2.88)	15.77–27.01	-	20.16 (3.81)	16.14–31.60	-	23.19(5.06)	17.09–35.42	-	*p* = 0.076
Family history of scoliosis	-	-	5 (27.78)	-	-	6 (33.33)	-	-	2(11.11)	*p* = 0.370
Comorbidities	-	-	3(16.67)	-	-	3 (16.67)	-	-	4(22.22)	*p* < 1
Physical education	-	-	13(72.00)	-	-	11(61.1)	-	-	17(94.44)	*p* = 0.054
Recreational sport activity [hours/week]	3.29 (1.38)	1–5	-	3.08 (1.78)	1–7	-	3.81 (3.16)	1–12	-	*p* = 0.899

Clinical and radiological characteristics (CBTSS, HFS)	CBTSS				CSS				p value comparison CBTSS/CSS (preoperatively/ postoperatively)	
	Preoperatively		Postoperatively		Preoperatively		Postoperatively			
	Mean (SD)	Range (Min-Max)	Mean (SD)	Range (Min-Max)	Mean (SD)	Range (Min-Max)	Mean (SD)	Range (Min-Max)		
Duration of CBT [hours]	5.22 (2.0)	2–9	5.80 (2.24)	1–9	--					
Duration of brace treatment prior to surgery [in months]	24.11 (20.20)	0–72	-		35.06 (25.43)	0–84	-		*p* = 0.162	
Cobb angle in the main curve	61.33 (8.0)	52–78	23.22 (7.69)	16–50	62.16 (10.70)	46–86	24.27 (8.76)	12–46	*p* = 0.793/*p* = 0.466	
Kyphosis angle in the thoracic spine	26.33 (19.11)	6–40	18.94 (7.99)	7–37	21.22 (11.31)	4–38	18.28 (7.58)	4–32	*p* = 0.419/*p* = 0.799	
Lordosis angle in the lumbar spine	48.61 (9.98)	24–70	39.28 (10.44)	12–64	38.33 (11.72)	15–60	30.59 (10.95)	6–55	*p* = 0.008*/*p* = 0.020*	
Apical translation of the central sacral vertical line (CSVL) according to the Harms Study Group) [cm]	4.43 (1.96)	0.3–8.7	1.51 (1.31)	0-5.8	4.97 (1.87)	1.3–8.6	1.68 (0.82)	0.5–3.2	*p* = 0.410/*p* = 0.254	
% of scoliosis correction	-		60.50 (11.67)	34–76	-		61.50 (11.46)	38–81	*p* = 0.949	

*CBT SS *CBT scoliosis sample, *CSS* Control scoliosis sample, *HFS* Healthy female sample

*p<0.05

The CBT intervention was delivered in an individualized manner, with total duration ranging from 1 to 9 h (mean 5.22, SD 2.0 h preoperatively and 5.8, SD 2.24 h postoperatively), depending on patients’ psychological needs and clinical availability.

According to the AIS Lenke Classification, type 1 was identified in the CBT scoliosis sample group in 12 patients (66.67), type 2 in 2 patients (11.11%), type 3 in 2 patients (11.11%) and type 6 in the remaining 2 patients (11.11%). In the control scoliosis sample, type 1 Lenke Classification, was indicated in 9 patients (50%), type 2 in 3 patients (16.67%), type 3 in 3 patients (16.67%) and type 6 in 3 patients (16.67%).

The additional data on all study groups are summarized in Table 1.

Referring to socio-demographic data, all study groups differed with regard to place of residence (*p* = 0.00002) only. Considering clinical and radiological data, scoliosis samples differ significantly in regards to preoperative and postoperative lordosis angle in the lumbar spine (*p* = 0.008 and *p* = 0.020, respectively).

Physical activity was comparable across the three groups. Specifically, participation in physical education classes did not differ significantly between groups (CBTSS: 72.2%; CSS: 61.1%; HFS: 94.4%; *p* = 0.054), nor did weekly hours of recreational sport (CBTSS: 3.29, SD 1.38; CSS: 3.08, SD 1.78; HFS: 3.81, SD 3.16; *p* = 0.899; see Table 1).

### Descriptive statistics of study measures

Patients in the CBT scoliosis sample scored 37.17 (SD 8.67) in STAIC-trait preoperatively, postoperatively 35.06 (SD 8.17), and 36.54 (SD 8.88) in the follow-up. Meanwhile, in the preoperative assessment, the control scoliosis sample scored 35.89 (SD 7.14), in the postoperative assessment the mean value was 36.72 (SD 0.28), whereas in the follow-up it was 36.20 (SD 2.04). In the healthy female sample, the mean STAIC- trait value was 41.72 (SD 10.50).

Regarding TAPS, patients in the CBT scoliosis sample scored 2.69 (SD 1.06), 1.31 (SD 0.42) and 1.33 (SD 0.33) preoperatively, postoperatively and in the follow-up, respectively. Patients in the control scoliosis sample scored 2.56 (SD 0.96), 1.22 (SD 0.28) and 1.46 (SD 0.53) preoperatively, postoperatively and in the follow-up, respectively. Meanwhile, the mean TAPS result obtained in healthy females was 1.37 (SD 0.42).

In reference to TRACE performed by the patients, the mean preoperative TRACE scores were 9.11 (SD 1.57) and 8.00 (SD 2.6) the CBT scoliosis sample and the control scoliosis sample, respectively. The postoperative TRACE scores were 3.22 (SD 2.58) and 2.33 (SD 1.68) in the CBT scoliosis sample and control scoliosis sample, respectively. In the follow-up, the TRACE scores were 2.08 (SD 1.55) and 3.09 (3.13) in the CBT scoliosis sample and the control scoliosis sample, respectively.

Regarding the TRACE performed by the doctors, the mean preoperative TRACE scores were 9.17 (SD 2.33) and 9.61 (SD 1.89) in the CBT scoliosis sample and control scoliosis sample, respectively. The postoperative TRACE scores were 2.67 (SD 1.71) and 2.11 (SD 1.57) in the CBT scoliosis sample and control scoliosis sample, respectively. In the healthy female sample, the mean TRACE value obtained by controls was 3.06 (SD 2.18) (Table 2).

**Table 2:** Descriptive statistics of STAIC-trait, TAPS and TRACE

	Mean (SD)	95% Confidence interval(from-to)	Range(Min-Max)		Mean (SD)		95% Confidence interval(from-to)	Range(Min-Max)	Mean (SD)	95% Confidence interval(from-to)	Range(Min-Max)
	Preoperatively				Postoperatively				Follow-up		
CBTSS											
STAIC-trait	37.17(8.67)	32.86–41.48	21.00–50.00		35.06(8.17)		31.00-39.12	20.00–47.00	36.54(8.88)	31.17–41.91	24.00–52.00
TAPS	2.69(1.06)	2.16–3.21	1.00–4.00		1.31(0.42)		1.11–1.52	1.00–2.00	1.33(0.33)	1.13–1.53	1.00–2.00
TRACE-patients	9.11(1.57)	8.33–9.90	5.0–11.0	3.22(2.58)		1.94–4.50		1.00–9.00	2.08(1.55)	1.14–3.06	1.00–5.00
TRACE-doctors	9.17(2.33)	8.01–10.33	3.0–12.00	2.67(1.71)		1.81–3.52		1.00–5.00	-		
CSS											
STAIC-trait	35.89(7.14)	32.33–39.44	22.00–44.00		36.72(0.28)		33.30-40.16	24.00–49.00	36.20 (2.04)	35.00–37.00	25.00–44.00
TAPS	2.56(0.96)	2.08–3.03	0.66–4.33		1.22(0.28)		1.08–1.36	1.00-1.67	1.46 (0.53)	1.00-1.67	1.00-2.67
TRACE-patients	8.00(2.6)	6.70–9.30	1.00–12.00		2.33(1.68)		1.50(3.17)	1.00–7.00	3.09 (3.13)	1.00–5.00	1.00–11.00
TRACE-doctors	9.61(1.89)	8.67–10.55	5.00–12.00		2.11(1.57)		1.33(2.89)	1.00–5.00	-		
HFS (one assessment)											
STAIC-trait	41.72(10.50)	36.50-46.95	25.00–59.00								
TAPS	1.37(0.42)	1.16–1.58	1.00-2.33								
TRACE-participants	3.06(2.18)	1.97–4.14	1.00–9.00								
TRACE-doctors	1.00(0.00)	-	1.00–1.00								

*CBT SS *CBT scoliosis sample, *CSS* Control scoliosis sample, *HFS* Healthy female sample, The State Trait Anxiety Inventory-trait (STAIC-trait), Trunk Appearance Perception Scale (TAPS), The Trunk Aesthetic Clinical Evaluation (TRACE), standard deviation (SD)

### Longitudinal analyses

Table 3. presents the results of longitudinal analyses referring to the questionnaire results in both scoliosis samples. The comparative analyses were planned as follows: preoperative/postoperative, /follow-up and postoperative follow-up.

**Table 3:** Longitudinal analyses in CBTSS and CSS

	Comparison preoperative/postoperative/follow-up					
	CBTSS			CSS		
STAIC- trait total score	*p* = 0.161			*p* = 0.575		
TAPS total score	Comparison preoperative/postoperative /follow-up					
	CBTSS			CSS		
	*p* = 0.0003*			0.023*		
	Comparison preoperative/postoperative: *p* = 0.018*	Comparison preoperative/follow-up: *p* = 1	Comparison preoperative/follow-up: *p* = 0.018*	Comparison preoperative/posoperative: *p* = 0.041*	Comparison postoperative/follow-up: *p* = 1	Comparison preoperative/follow-up: *p* = 0.353
TRACE-patients	Comparison preoperative/postoperative /follow-up					
	CBTSS			CSS		
	*p* = 0.00002*			*p* = 0.0006*		
	Comparison preoperative/postoperative *p* = 0.004*	Comparison postoperative /follow-up: *p* = 1	Comparison preoperative /follow-up: *p* = 0.0001*	Comparison preoperative / postoperative: *p* = 0.002*	Comparison postoperative /follow-up: *p* = 1	Comparison preoperative /follow-up: *p* = 0.023*
TRACE-doctors (preoperative/postoperative)	CBTSS	CSS	-			
	*p* = 0.0002*	*p* = 0.0002*				

*CBT SS *CBT scoliosis sample, *CSS* Control scoliosis sample, *HFS* Healthy female sample, The State-Trait Anxiety Inventory-trait (STAIC-trait), Trunk Appearance Perception Scale (TAPS), The Trunk Aesthetic Clinical Evaluation (TRACE)

*p<0.05

Regarding both scoliosis samples, in the case of the STAIC-trait, the results of comparisons between the three assessment times, did not reveal any significant differences (*p* = 0.161 and *p* = 0.575 for the CBT scoliosis sample and the control scoliosis sample, respectively).

In regard to TAPS, the differences between all assessments were significant for both scoliosis groups, in favor, as expected, of postsurgical results. However, the improvement of the perception of patients’ body shape remained significant for the presurgical-follow-up comparison (*p* = 0.018) only in the CBT scoliosis sample.

In regard to TRACE (patients) the differences were significant for all comparisons in both scoliosis samples, similarly, as in TAPS, in favor of postsurgery evaluations. However, the improvement of body shape perception remained significant in the follow-up in both scoliosis samples.

In regard to TRACE (doctors) the difference were, as expected, significant for pre- and postsurgical comparison in both scoliosis samples (*p* = 0.002 and *p* = 0.002). For details, see Table 3.

### Cross-sectional analyses

Table 4. presents results of preoperative, postoperative and follow-up cross-sectional analyses. The comparative analyses were planned as follows: comparison between both scoliosis samples, comparison between the CBT scoliosis sample and the healthy female sample and between the scoliosis control sample and the healthy female sample. In addition, to verify, if scoliosis samples are in accordance with their doctors perceptions’ of their bodies, Kendall’s coefficient of concordance ( Kendall’s W) was calculated, both pre-and postsurgery.

**Table 4:** Cross-sectional analyses between CBTSS, CSS and HFS

	Comparison CBTSS/CSS/HFS		
Preoperative STAIC-trait	*p = 0.127*		
Preoperative STAIC-trait	*p* = 0.513		
Follow-up STAIC-trait	*p* = 0.130		
Preoperative TAPS Total score	*p* = 0.001*		
Preoperative TAPS total score: results of post-hoc tests	CBTSS/CSS *p* = 1.00	CBTSS/HFS *p* = 0.0005*	CSS/HFS *p* = 0.001*
Postoperative TAPS	*p* = 0.69		
Follow-up TAPS	*p* = 0.890		
Preoperative TRACE-patients	*p* < 0.0000001*		
Preoperative TRACE-patients: results of post-hoc tests	CBTSS/CSS *p* = 0.745	CBTSS/HFS *p* = 0.000001*	CSS/HFS *p* = 0.0002*
Postoperative TRACE-patients	*p* = 0.377		
Follow-up TRACE-patients	*p* = 0.224		
Preoperative TRACE-doctors	*p* < 0.00001		
Preoperative TRACE-doctors: results of post-hoc tests	CBTSS/CSS *p* = 1	CBTSS/HFS *p* < 0000001	CSS/HFS *p* = 000002
Postoperative TRACE-doctors	*p* = 0.313		
	Comparison: TRACE-patients/TRACE-doctors: Kendall’s coefficient of concordance ( Kendall’s W)		
Preoperative TRACE in CBTSS	Kendall’s W = 0.542		
Postoperative TRACE in CBTSS	Kendall’s W = 0.341		
Preoperative TRACE in CSS	Kendall’’s W = 0.380		
Postoperative TRACE in CSS	Kendall’s W = 0.508		

*CBT SS *CBT scoliosis sample, *CSS* Control scoliosis sample, *HFS* Healthy female sample, The State-Trait Anxiety Inventory-trait (STAIC-trait),  Trunk Appearance Perception Scale (TAPS), The Trunk Aesthetic Clinical Evaluation (TRACE)

*p<0.05

In relation to comparison between all study samples, the study groups differed in regard to preoperative results only: TAPS (*p* = 0.001), TRACE-patients (*p* < 0.0000001) and TRACE-doctors (*p* < 0.00001). Then, the multiple comparisons were made. The significant differences, as expected, referred to comparisons of both study samples with healthy controls (for details see Table 4). In regards to follow-up results, any significant differences between all study groups were identified.

Referring to TRACE-patients and TRACE-doctors results agreement, we indicated there is no compliance between them (results of Kendall’s coefficient of concordance appeared insignificant, for details see Table 4).

### Correlational analyses: relations between anxiety levels and body perception in scoliosis samples

Table 5 presents the results of correlational analyses between patients’ and doctors’ evaluation of body shape, and anxiety levels. Significant associations were revealed in the CBT scoliosis sample between TAPS and STAIC in the postsurgical and follow-up assessment only (rs = 0.50 and rs = 0.59, respectively).

**Table 5:** Correlational analyses between STAIC-trait, TRACE, and TAPS in scoliosis samples

		CBTSS			CSS		
		TRACE-patients	TRACE-doctors	TAPS	TRACE-patients	TRACE-doctors	TAPS
	Preoperatively						
STAIC-trait		rs = 0.05	rs=-0.02	rs = 0.39	rs = 0.31	rs=-0.05	rs = 0.01
	Postoperatively						
STAIC-trait		rs = 0.22	rs=-0.20	rs = 0.50*	rs=-0.24	rs=-0.40	rs = 0.34
	Follow-up						
STAIC-trait		rs = 0.16	-	rs = 0.59*	rs = 0.12	-	rs = 0.43

*CBT SS *CBT scoliosis sample, *CSS* Control scoliosis sample, *HFS* Healthy female sample, The State-Trait Anxiety Inventory-trait (STAIC-trait), Trunk Appearance Perception Scale (TAPS), The Trunk Aesthetic Clinical Evaluation (TRACE)

*p<0.05; Spearman’s rank correlation coefficient (rs)

## Discussion

In this project, due to the longitudinal analysis of changes in anxiety levels and assessment of body deformity in AIS patients who either did or did not undergo CBT, new knowledge in the area of guidelines for psychological interventions for adolescents undergoing spinal surgery, was provided. In particular, the significance of CBT aimed at supporting AIS patients in accepting their postsurgical body shape, and for their anxiety levels during the hospital stay and in the long-term, was analyzed.

In addition, in the current study, it was assumed that the emotional state, especially anxiety and tension, may be related to patients’ assessment of spinal appearance. Thus, we accepted it was necessary to investigate the emotional burden females with AIS experience due to spinal disfigurement and due to surgical treatment, in order to provide them with the most suitable kind of psychological support. From the clinical point of view, it is essential to increase the compliance of adolescent patients with the requirements connected with the post-surgical period, and, thus, for a successful treatment outcome.

It must be emphasized, surgical treatment of scoliosis is regarded as the pediatric orthopedic operation carried out that places the most strain on the patient’s body [20]. It was previously revealed that adolescent patients are anxious presurgery mainly about the risk of complications. In particular, patients’ concerns regard the possibility of damage to the spinal cord. On the other hand, the period of convalescence and return to full health is very long, and at the same time, changes patients’ habits due to the necessity for adherence to the recommendations of the doctor [21].

Little is still known about changes in AIS patients’ assessment of spinal appearance across a longer time-frame perspective, as well as about relations between anxiety levels and selected cognitive functions or perceptions (perhaps overestimation? ) of spinal appearance in particular. For example, Noonan et al. [2] found that the perception of self-image deteriorates with duration of follow-up, however this association was especially distinct in scoliosis patients treated operatively as compared to patients treated conservatively. On the other hand, Głowacki et al. [6] reported that in patients treated conservatively, body shape perception and anxiety levels remained stable over time. Their analysis of the Spinal Appearance Questionnaire (SAQ) further indicated improvement in the evaluation of the spinal curve, particularly after 12 months of brace treatment.

Meanwhile, in the current study, the longitudinal, threefold assessment of body image was based on detailed, self-evaluation of views of the trunk from behind and in the axial plane as well as a frontal view, which probably corresponds to the most realistic perception of one’s body [14]. Such analysis is of particular importance in the prevention of persistence of body-image disturbances in the long-term following scoliosis surgical treatment. Interestingly, TAPS results indicated an immediate improvement in body shape perception after surgery in both scoliosis groups. However, what is especially important in the clinical context, the scoliosis control sample did not maintain significant improvement of body shape perception in the follow-up evaluation, when compared to the preoperative evaluation. Only in CBT scoliosis sample, did the improvement of body image remain stable in the follow-up.

It should be noted that adolescents’ subjective perception of trunk deformity may be influenced not only by the objective severity of spinal curvature, but also by lifestyle factors, such as participation in sports, and by individual body awareness [22]. This suggests that improvements in body shape perception following surgery, as captured by TAPS, may reflect both structural changes and psychosocial factors related to patients’ engagement in physical activities and their own perception of body aesthetics. Considering these factors helps contextualize our TAPS findings and supports the need for individualized psychological interventions to promote accurate and positive body perception.

Interestingly, accounting for TRACE-patients results, it must be highlighted, that the improvements in body shape perceptions appeared to be stable in the follow-up. However, this assessment tool is dedicated particularly to doctors, to help them gain an aesthetic evaluation of scoliosis patient trunk deformity. This is the first use of TRACE in the patient population. As we indicated above, both scoliosis samples were asked to complete it, in order to calculate the possible inaccuracy in the assessment of body disfigurement between patients and their doctors. Interestingly, due to this procedure, lack of agreement between patients’ and doctors’ evaluation of body shape, independently from received therapeutic support, and both pre- and Postsurgery, was revealed. As follow-up data were collected only from patient groups (with the third assessment conducted by mail), it was not possible to determine whether these discrepancies remained stable after surgery or whether they were associated with the received psychological support. This issue should be verified perhaps in a longer follow-up, during a medical check-up.

Essau et al. [23] found that adolescent females between the ages of 12 and 17 exhibit more anxiety symptoms than males and that this symptom tends to increase with age. Moreover, it was indicated that clinically significant body image concerns or body image disorders are associated with higher levels of depression, anxiety or mood disorders in general [24]. Also Mori et al. [25] pointed out that dissatisfaction with body image may play a significant role in the development of low self-esteem, emotional problems and depression. Meanwhile, Głowacki et al. [6] supported the evidence of relationships between patients’ perception of Trunk shift and anxiety, which indicated AIS patients’ subjective evaluation of body appearance might be influenced by their emotional states. Interestingly, this association took place at the beginning of the brace treatment period only. In the current study, significant moderate relations between anxiety levels and body shape perception were confirmed in the follow-up in the CBT group only, the higher anxiety level was associated with more negative perceptions of body shape. This might mean that psychological support should be tailored individually in a longer follow-up, especially to those patients who remain unsatisfied with the results of surgical treatment, irrespective of objective, good clinical and radiological treatment outcomes. On the other hand, patients who experience emotional strain, e.g., high anxiety, irrespective of the scoliosis diagnosis and applied treatment methods, also might still overestimate perception of body disfigurement, even in a longer follow-up.

### Strengths and limitations

The novel aspect of this research was the combination of a 3-fold, longitudinal assessment as well as the cross-sectional research strategy. Thus, surgical outcomes could be evaluated from multiple perspectives: those of adolescents with scoliosis, healthy adolescent females, and clinicians. At the same time, the efficacy of CBT interventions for patients’ emotional state and body image was evaluated.

However, some limitations of the present study should be noted. Firstly, the sample size (*n* = 36 in total) was relatively small. Another limitation is the attrition observed at the final follow-up assessment (T3), particularly in the scoliosis control group, which reduced the sample size and may have introduced bias in the longitudinal analyses. This attrition could limit the generalizability of the findings and reduce statistical power, and therefore the results of the follow-up evaluation should be interpreted with caution.

Furthermore, this study focused exclusively on females with thoracic scoliosis, which may have influenced score distributions and limited the generalizability of the findings.

What is more, the individualized nature of the CBT intervention, including variability in total duration and mode of post-discharge contact, reflects routine clinical practice but may have contributed to variability in treatment response. As the study was not designed to examine dose–response relationships, this should be considered a limitation.

Additionally, the current study did not address other psychological variables, that might have influenced anxiety levels, e.g., depression, neuroticism, emotion regulation skills or some variables connected with a family system. Thus, to expand the generalizability of the current study, we postulate future studies would examine, e.g., males and females with AIS in a longer than 6 months follow-up after scoliosis surgery.

### Clinical implications

The findings highlight the potential value of integrating structured, individualized CBT into the surgical care pathway for adolescents with scoliosis. CBT may support the maintenance of positive body image postoperatively and help adolescents cope with anxiety related to appearance-specific concerns. Interventions should be considered both pre- and postoperatively, with particular attention to patients who continue to perceive their body negatively despite successful surgical outcomes. Implementing such psychological support could enhance patient well-being, improve adherence to postoperative recommendations, and potentially optimize overall treatment outcomes.

## Conclusions

Conservative or surgical scoliosis treatment can be regarded by young patients as a difficult, stressful and threatening experience. Results of the current study indicate that perceptions of patients’ body shape improve immediately after surgery. However, long-term stabilization of body image appears to benefit from psychological support. Anxiety levels remained stable across the follow-up period, independent of received therapeutic support. Patients with persistent negative appearance-specific cognitions may require targeted, individualized psychological interventions. 

## Data Availability

The datasets used and/or analysed during the current study are available from the corresponding author on reasonable request.
